# Corneal Infection Therapy with Topical Bacteriophage Administration

**DOI:** 10.2174/1874364101509010167

**Published:** 2015-11-04

**Authors:** Ali Fadlallah, Elias Chelala, Jean-Marc Legeais

**Affiliations:** 1 Pierre et Marie Curie University, Sorbonne Universities, Paris, France; 2 Paris Descartes University, Paris, France; 3 Hôtel Dieu de Paris Hospital, Paris, France; 4 Saint-Joseph university, Faculty of Medicine, Beirut, Lebanon

**Keywords:** VRSA, Interstitial Keratitis, phage therapy.

## Abstract

Staphylococcus aureus is a major pathogen in bacterial keratitis, a vision-threatening disease. Although the incidence of S. aureus keratitis varies worldwide, the increasing trend of resistance to certain antibiotics makes this condition an important, global, healthcare concern. We report the case of a 65-year-old woman with nosocomial left-eye corneal abscess and interstitial keratitis.The patient then undergo topical Phage therapy with successful results.

## INTRODUCTION 

*Staphylococcus aureus* is a major pathogen in bacterial keratitis, a vision-threatening disease [1]. The most common manifestations of ocular *S. aureus* infections are conjunctivitis, keratitis, and/or eyelid disorders.

Although the incidence of *S. aureus* keratitis varies worldwide, the increasing trend of resistance to certain antibiotics makes this condition an important, global, healthcare concern [2]. Methicillin-resistant *S. aureus* (MRSA) infection first emerged in the United States in the 1970s, and by the 1990s, MRSA was considered endemic in most large medical centers [3]. Glycopeptides are the antibiotics of choice in treating MRSA infections. The first strain of *S. aureus* with reduced susceptibility to vancomycin and teicoplanin was reported in Japan, whereas the first isolate of vancomycin-resistant *S. aureus* (VRSA) was reported in healthy carriers in Brazil [4, 5]. Therefore, the development of new adjunctive or alternative therapies for the treatment of bacterial keratitis is warranted.

Since the 1980s, phage therapy has been reevaluated in Western countries. As the dilemma of antibiotic resistance grows, new antimicrobial strategies must be found or our healthcare system will revert to a preantibiotic era for many pathogens. This has become a major priority of WHO, as well as politicians and public health systems around the world [6]. Antibacterial agents against which resistance has not yet evolved, ones that are inexpensive and also display low toxicities are needed. Bacteriophages, in particular, exhibit these characteristics. The therapeutic efficacy of phage therapy *in vivo* has been demonstrated in several murine models of antibiotic-resistant bacterial infection, including MRSA [6].

**Fig. (1):**
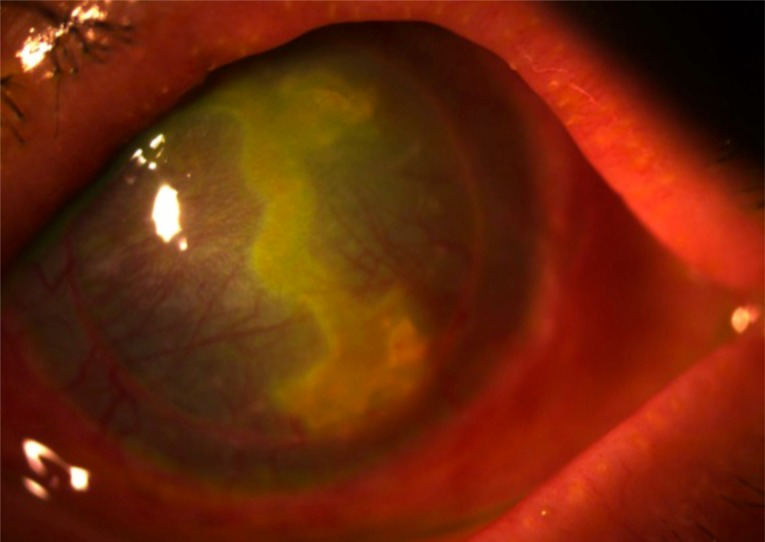
Slit Lamp photo showing active bacterial keratitis.

We report the case of a 65-year-old woman who underwent craniotomy for acoustic neurinoma, complicated by a postoperative left-eye corneal abscess and interstitial keratitis (no facial paralysis, or lagophthalmos developed then after). Nosocomial MRSA infection was identified at the time, and her condition resolved with administration of 50 mg/mL fortified vancomycin. Subsequently, she underwent penetrating keratoplasty (PKP) in the left eye for the post infectious corneal scarring. Three months later, the patient was diagnosed with a corneal abscess and started on broad-spectrum topical antibiotics. Cultures were positive for vancomycin-intermediate sensitivity *S. aureus* (VISA). Several days later, when substantial corneal thinning was observed, we applied an amniotic membrane graft and prescribed oral doxycycline 50 mg twice daily. The vancomycin dosage was increased, thereby resulting in lessening of the infiltrate. At week 3 of treatment, topical cyclosporine 0.5% started four times daily for treatment of residual stromal inflammation. With this regimen, the ulcer gradually healed with a stromal scar. The patient was maintained on oral pristanamycine for chronic portage of *S. aureus* infection (manifested by chronic dermatitis, blepharitis and rhinosinusitis). The patient was given nasal mupirocin twice daily for 5 days and instructed to wash her hands and body with chlorhexidine gluconate 4% soap, to reduce the possible sources and spread of pathogenic staphylococci. A PKP was repeated 8 months later owing to the recurrence of corneal abscess and interstitial keratitis; microbial cultures were positive for VRSA. This condition lasted for another 11 years with persistent positive cultures revealing chronic nasal, dermatological, and ocular VISA carriage (Fig. **1**).

The patient then decided to undergo Phage therapy at a specialized center in Georgia (Phage Therapy Center, Tbilisi, Georgia) using the *S*. *aureus* bacteriophage SATA-8505 (ATCC PTA-9476) during 4 weeks. *S*. *aureus* bacteriophage SATA-8505 (ATCC PTA-9476) was examined for its ability to prevent and treat infections with clinically relevant MRSA strain USA300 *in vitro* in human cells and *in vivo* in mice (Patent no: US 7,745,194 B2). SATA-8505 is a model phage because of its commercial availability and patented claims of activity against USA300 *in vitro* with classifications 435/235.1 (USA), C12P1/06 (International), C12N7/00, C12N2795/00021 (Cooperative), and C12N7/00 (European) (Patent no: US 7,745,194 B2). Patient underwent a topical (eye drops and nasal spray) and general (intravenous) Phage therapy treatment against resistant S. aureus.

**Fig. (2):**
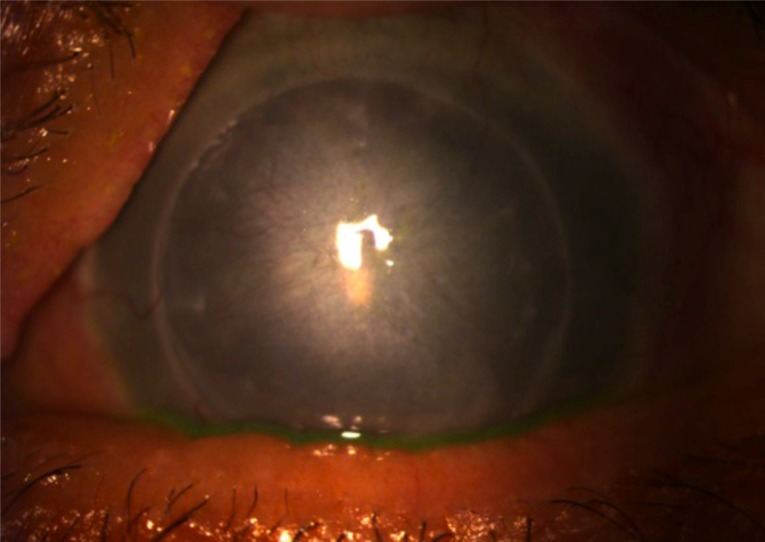
Slit Lamp photo showing cornea 3 months after topical bacteriophage administration.

Three and six months after treatment, she was examined at our center with a stabilization of her ocular signs, and a negative ocular and nasal culture confirmed by 2 independent laboratories (Hotel-Dieu, Paris, France and Labazur, Paris, France) (Fig. **2**). 

This case indicates that bacteriophage eye-drops may be a novel adjunctive or alternative therapeutic agent for the treatment of infectious keratitis secondary to antibiotic-resistant bacteria. As the incidence of bacterial keratitis is increasing because of inappropriate use of soft contact lenses and infection with multidrug-resistant bacteria, clinical trials are warranted to assess the therapeutic potential of phages in ocular disease, particularly in antibiotic-resistant cases.
